# 2678. Prevalence of gonorrhea, chlamydia and syphilis among males living with HIV attending the Antiretroviral Clinic, Hospital Santo Tomás, Panamá, November 2021-March 2022

**DOI:** 10.1093/ofid/ofad500.2289

**Published:** 2023-11-27

**Authors:** Cristel Rodriguez, Ana Belén Araúz Rodríguez, Amanda Gabster, Monica Jhangimal, German Henostroza, Juan Miguel Pascale, Jorge Castillo, Alexander Martinez, Alma Ortiz

**Affiliations:** Hospital Santo Tomás, Panama, Panama, Panama; Hospital Santo Tomás, Panama, Panama, Panama; Gorgas Memorial Institute for Health Studies, Panama, Panama, Panama; Gorgas Memorial Institute for Health Studies, Panama, Panama, Panama; William Crawford Gorgas Center for Geographic Medicine, University of Alabama at Birgminham, Birmingham, Alabama, Alabama, Alabama; Gorgas Memorial Institute for Health Studies, Panama, Panama, Panama; Gorgas Memorial Institute for Health Studies, Panama, Panama, Panama; Gorgas Memorial Institute for Health Studies, Panama, Panama, Panama; Gorgas Memorial Institute for Health Studies, Panama, Panama, Panama

## Abstract

**Background:**

Prevalence of gonorrhea, chlamydia and syphilis is unknown among people living with HIV (PLHIV) in Panama. Owing to the asymptomatic course and syndromic management of these infections, etiological diagnosis and epidemiological surveillance are often missed. The aim of the study was to determine the prevalence of *Neisseria gonorrhoeae* (NG), *Chlamydia trachomatis* (CT) and syphilis in HIV positive men, that receive care in the Infectious Diseases Clinic at Hospital Santo Tomás.

**Methods:**

A cross-sectional study was conducted among male PLHIV who attended the antiretroviral therapy clinic for a routine clinical care during March and April 2022. Written consent was obtained from all participants. Blood samples were taken for syphilis screening using a rapid lateral flow test and consecutive RPR on all positive samples. First flow urine, as well as provider-collected oropharyngeal and participant-collected anal swabs were taken from the patients for PCR CT/NG testing. Statistical analysis was carried out using SPSS version 22.0 software. Chi-square test and odds ratio were done at 95% CI using significance level of p < 0.05.

**Results:**

A total of 221 male PLHIV were included. The prevalence of CT/NG at any anatomical site was 20.3%, 13.5% for CT and 9.0% for NG. The anatomical site with the highest prevalence was the rectal site, with a prevalence of 12.2% for CT/NG. *Neisseria gonorrhoeae* had a prevalence of 5.4% in oropharyngeal samples. An antibody syphilis test was positive in 51.1% of the patients. Logistic regression analysis revealed that men that reported to have had sex with another male in the past year was a risk factor for CT/NG positivity at any site(AOR = 4.95, 95% CI 1.46-16.76, p 0.004). Overall, 91.4 % of the patients had good acceptance for auto collection of the rectal sample among the study group.

**Conclusion:**

There is a high prevalence of CT/NG, especially at the rectum site, and high prevalence of syphilis among HIV male atients in the antiretroviral clinic at the Santo Tomás Hospital in Panama. Most individuals with an STI w tested for, reported to be asymptomatic for these infections. Public health strategies need to be implemented to increase the screening and appropiate treatment with this population.

**Disclosures:**

**All Authors**: No reported disclosures

